# Daratumumab monotherapy for patients with intermediate-risk or high-risk smoldering multiple myeloma: a randomized, open-label, multicenter, phase 2 study (CENTAURUS)

**DOI:** 10.1038/s41375-020-0718-z

**Published:** 2020-02-05

**Authors:** C. Ola Landgren, Ajai Chari, Yael C. Cohen, Andrew Spencer, Peter Voorhees, Jane A. Estell, Irwindeep Sandhu, Matthew W. Jenner, Catherine Williams, Michele Cavo, Niels W. C. J. van de Donk, Meral Beksac, Philippe Moreau, Hartmut Goldschmidt, Steven Kuppens, Rajesh Bandekar, Pamela L. Clemens, Tobias Neff, Christoph Heuck, Ming Qi, Craig C. Hofmeister

**Affiliations:** 1 Department of Medicine, Myeloma Service, Memorial Sloan Kettering Cancer Center, New York, NY USA; 2 Icahn School of Medicine at Mount Sinai, New York, NY USA; 3 Department of Hematology, Tel-Aviv Sourasky (Ichilov) Medical Center, and Sackler School of Medicine, Tel Aviv University, Tel Aviv, Israel; 4 Malignant Haematology and Stem Cell Transplantation Service, Alfred Health-Monash University, Melbourne, VIC Australia; 5 Levine Cancer Institute/Atrium Health, Charlotte, NC USA; 6 Haematology Department, Concord Cancer Centre, Concord Hospital, University of Sydney, Concord, NSW Australia; 7 Department of Medicine, University of Alberta, Edmonton, AB Canada; 8 Southampton General Hospital, Southampton, UK; 9 Department of Clinical Haematology, Nottingham University Hospitals, Nottinghamshire, UK; 10 Department of Experimental, Diagnostic and Specialty Medicine, “Seràgnoli” Institute of Hematology, University of Bologna, Bologna, Italy; 11 Department of Hematology, VU University Medical Center, Amsterdam, The Netherlands; 12 Department of Hematology, Ankara University, Ankara, Turkey; 13 University Hospital Hôtel-Dieu, Nantes, France; 14 University Hospital Heidelberg and National Center of Tumor Diseases (NCT), Heidelberg, Germany; 15 Janssen Research & Development, Beerse, Belgium; 16 Janssen Research & Development, LLC, Spring House, PA USA; 17 Department of Hematology & Oncology, Winship Cancer Institute of Emory University, Atlanta, GA USA

**Keywords:** Myeloma, Molecularly targeted therapy

## Abstract

Current guidelines for smoldering multiple myeloma (SMM) recommend active monitoring until the onset of multiple myeloma (MM) before initiating treatment or enrollment in a clinical trial. Earlier intervention may delay progression to MM. In CENTAURUS, 123 patients with intermediate-risk or high-risk SMM were randomly assigned to daratumumab 16 mg/kg intravenously on extended intense (intense), extended intermediate (intermediate), or short dosing schedules. At the prespecified primary analysis (15.8-month median follow-up), the complete response (CR) rates (co-primary endpoint) were 2.4%, 4.9%, and 0% for intense, intermediate, and short dosing, respectively; the co-primary endpoint of CR rate >15% was not met. Progressive disease (PD)/death rates (number of patients who progressed or died divided by total duration of progression-free survival [PFS] in patient-years; co-primary endpoint) for intense, intermediate, and short dosing were 0.055 (80% confidence interval [CI], 0.014–0.096), 0.102 (80% CI, 0.044–0.160), and 0.206 (80% CI, 0.118–0.295), respectively, translating to a median PFS ≥24 months in all arms (*P* *<* 0.0001, <0.0001, and =0.0213, respectively). With longer follow-up (median follow-up, 25.9 months), CR rates were 4.9%, 9.8%, and 0% for intense, intermediate, and short dosing, respectively. PD/death rates for intense, intermediate, and short dosing were 0.059 (80% CI, 0.025–0.092), 0.107 (80% CI, 0.058–0.155), and 0.150 (80% CI, 0.089–0.211), respectively, again translating to a median PFS ≥ 24 months in all arms (*P* *<* 0.0001 for all arms). Twenty-four–month PFS rates were 89.9% (90% CI, 78.5–95.4%), 82.0% (90% CI, 69.0–89.9%), and 75.3% (90% CI, 61.1–85.0%) for intense, intermediate, and short dosing, respectively. Pharmacokinetic analyses indicated that intense dosing maintained target-saturating trough concentrations in most patients throughout weekly, every-2-week, and every-4-week dosing periods. No new safety signals were observed. These data provide the basis for an ongoing phase 3 study of daratumumab in SMM.

## Introduction

Multiple myeloma (MM) evolves from a premalignant, asymptomatic precursor stage known as smoldering MM (SMM) [1, 2]. Although patients are at variable risk for progression to MM, there is currently no uniformly accepted definition of high-risk or intermediate-risk SMM [1]. The previous Mayo Clinic model defined risk categories on the basis of three criteria: serum monoclonal protein (M-protein) ≥3 g/dl, clonal bone marrow plasma cells (PCs) ≥10%, and serum immunoglobulin free light chain (FLC) ratio <0.125 or >8. Patients meeting one, two, or three of these criteria are considered to be at low, intermediate, or high risk for progression to MM, respectively, with 5-year progression rates of 25%, 51%, and 76%, respectively [3]. The newer Mayo Clinic criteria use M-protein >2 g/dl, clonal bone marrow PCs >20%, and serum immunoglobulin FLC ratio >20 to define risk categories with 5-year risks for developing MM of 23% (low risk), 47% (intermediate risk), and 82% (high risk) [4]. In the PETHEMA model, risk categories are defined on the basis of two criteria: ≥95% abnormal PCs in the bone marrow, as assessed by multiparameter flow cytometry, and the presence of immunoparesis, defined as a decrease (below lower normal limit) in the levels of one or two immunoglobulins relative to those of the corresponding uninvolved immunoglobulin. Patients meeting one or two of these criteria were found to be at intermediate or high risk for progression to MM (5-year progression risks of 46% and 72%), respectively, and patients meeting neither of these criteria were found to be at low risk (5-year progression risk of 4%) [5].

Current standard of care for patients with SMM is active monitoring until progression to MM before initiating treatment [1]. However, most high- or intermediate-risk SMM patients do progress to MM [3, 5]. The phase 3 QuiRedex study of lenalidomide/dexamethasone (Rd) in patients with SMM, which was conducted before the definition of MM was revised to include validated biomarkers to allow earlier MM diagnosis [6], showed that Rd not only decreased the proportion of patients who developed MM (39 vs. 86%), but also improved overall survival (OS), albeit with treatment-related toxicities (one patient died due to treatment) [7, 8]. These findings suggest that intercepting high-risk or intermediate-risk SMM may yield clinical benefit, and that finding a less toxic intervention is an unmet need.

Daratumumab is a human IgG1κ monoclonal antibody that targets CD38, a receptor that is highly expressed on the myeloma cell surface [9, 10]. Daratumumab’s antitumor effects result from its on-tumor and immunomodulatory mechanism of action [11–16]. In the GEN501 and SIRIUS studies, daratumumab monotherapy induced deep and durable responses and had a favorable safety profile in patients with heavily pretreated relapsed and/or refractory MM (RRMM) [17, 18]. Based on these findings, we hypothesized that daratumumab monotherapy could delay progression from intermediate-risk or high-risk SMM to MM compared with historical observations [19]. We therefore initiated this phase 2 study of daratumumab monotherapy in patients with intermediate-risk or high-risk SMM.

## Patients and methods

### Study design

This was a randomized, open-label, multicenter, phase 2 study in patients with high-risk or intermediate-risk SMM (ClinicalTrials.gov Identifier: NCT02316106). All patients provided written informed consent. The study was approved by institutional review boards or ethics committees at all participating institutions and was conducted in accordance with the Declaration of Helsinki and the International Conference on Harmonisation Good Clinical Practice guidelines.

### Patients

Eligible patients ≥18 years of age had a confirmed diagnosis of high-risk or intermediate-risk SMM for <5 years, and an Eastern Cooperative Oncology Group (ECOG) performance status score of 0 or 1. High-risk or intermediate-risk SMM was defined as bone marrow PCs ≥10% to <60% and at least one of the following: serum M-protein ≥3 g/dl (IgA ≥2 g/dl), urine M-protein >500 mg/24 h, abnormal FLC ratio (<0.126 or >8) with serum M-protein <3 g/dl but ≥1 g/dl, or (criteria added following a protocol amendment) absolute involved serum FLC ≥100 mg/l with an abnormal FLC ratio (<0.126 or >8, but not ≤0.01 or ≥100). Key exclusion criteria included the presence of at least one SLiM-CRAB myeloma-defining event, as defined in the 2014 International Myeloma Working Group (IMWG) criteria (≥60% bone marrow PCs, FLC involved/uninvolved ratio ≥100, >1 focal bone lesion by magnetic resonance imaging [MRI], calcium elevation, renal insufficiency by creatinine clearance, anemia, or bone disease due to lytic bone lesions) [6], pretreatment clinical laboratory values indicating clinically relevant organ dysfunction (absolute neutrophil count <1.0 × 10^9^/l, platelet count <75 × 10^9^/l, aspartate aminotransferase and alanine aminotransferase >2.5 × the upper limit of normal, and total bilirubin >1.5 × the upper limit of normal), and primary systemic AL (immunoglobulin light chain) amyloidosis. Patients were also excluded for concurrent bisphosphonate treatment for SMM or MM.

### Treatments

**Fig. 1:**
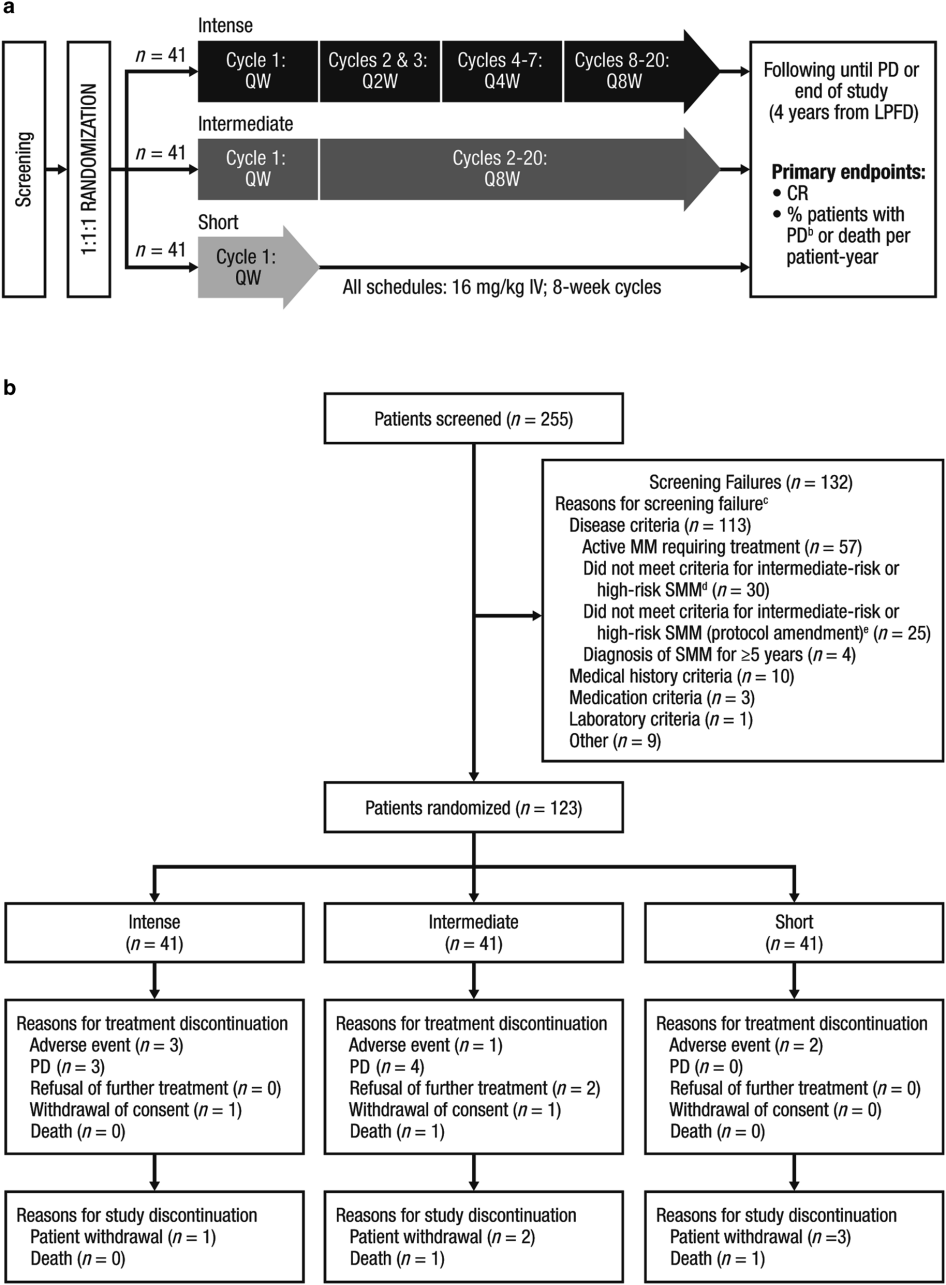
Study design and patient flow diagram. **a** Study design. **b** Patient flow diagram through the clinical cutoff date.^a^ *QW* once weekly, *Q2W* every 2 weeks, *Q4W* every 4 weeks, *Q8W* every 8 weeks, *IV* intravenously, *PD* progressive disease, *LPFD* last patient first dose, *CR* complete response, *MM* multiple myeloma, *SMM* smoldering multiple myeloma, *IMWG* International Myeloma Working Group, *FLC* free light chain, *PC* plasma cell. ^a^June 29, 2018. ^b^PD was defined per the 2014 *IMWG* criteria for MM [6] plus additional *IMWG* FLC progression criteria (a ≥25% increase from nadir in the difference between involved and uninvolved FLC levels [absolute increase must be >10 mg/dl]) [21]. ^c^A patient could have multiple reasons for exclusion and therefore be counted in more than one category. ^d^Bone marrow PCs ≥10% to <60% plus serum M-protein ≥3 g/dl (IgA ≥2 g/dl), urine M-protein >500 mg/24 h, or abnormal FLC ratio (<0.126 or >8) and serum M-protein <3 g/dl but ≥1 g/dl. ^e^Bone marrow PCs ≥10% to <60% plus serum M-protein ≥3 g/dl (IgA ≥2 g/dl), urine M-protein >500 mg/24 h, abnormal FLC ratio (<0.126 or >8) and serum M-protein <3 g/dl but ≥1g/dl, or absolute involved serum FLC ≥100 mg/l with an abnormal FLC ratio (<0.126 or >8, but not ≤0.01 or ≥100).

Patients (*N* = 123) were randomly assigned (1:1:1 ratio) using an interactive web-based system to receive one of three daratumumab dosing schedules: extended intense (intense), extended intermediate (intermediate), and short (Fig. 1a). Randomization was balanced using permuted blocks (block size 6) and was stratified based on number of risk factors for progression to MM (<2 vs. ≥2; Supplementary Methods) [20]. Daratumumab 16 mg/kg was administered intravenously in 8-week cycles. In the intense arm, patients received daratumumab weekly in Cycle 1, every 2 weeks in Cycles 2 and 3, every 4 weeks in Cycles 4–7, and every 8 weeks in Cycles 8–20. In the intermediate arm, patients received daratumumab weekly in Cycle 1 and every 8 weeks in Cycles 2–20. The every-8-week dosing schedule used in Cycles 8–20 in the intense arm and Cycles 2–20 in the intermediate arm was selected for patient convenience. In the short arm, patients received one cycle of weekly daratumumab. Patients were followed until disease progression and will be monitored until the end of the study (4 years from the time the last patient received the first daratumumab dose). Treatment assignments were not blinded.

### Endpoints and assessments

The co-primary endpoints were complete response (CR) rate and progressive disease (PD)/death rate per patient-year. The primary analysis of CR rate, defined as the proportion of patients who achieved ≥CR (CR or stringent CR [sCR] by IMWG criteria [21, 22]), was conducted 6 months after the last patient was randomized. The primary analysis of PD/death rate, defined as the number of patients who progressed to MM or died divided by the total duration of progression-free survival (PFS) for all patients, in patient-years (with PD defined per 2014 IMWG [SLiM-CRAB] criteria [6] plus additional IMWG FLC progression criteria [in addition to an involved/uninvolved serum FLC ratio of ≥100 and an involved FLC of ≥100 mg/l, a ≥25% increase from nadir in the difference between involved and uninvolved FLC levels, with an absolute increase of >10 mg/dl [6, 21], all of which had to be met during two consecutive visits]; Supplementary Table S1), was conducted 12 months after the last patient was randomized. The PD/death rate cutoff chosen for this endpoint—a PD/death rate of ≥0.346/patient-year as the null hypothesis—translates to a median PFS cutoff of 24 months, assuming an exponential survival function; the null hypothesis was that patients will have a median PFS <24 months. The PFS cutoff of 24 months was selected based on a prior study [19]. Secondary endpoints included overall response rate (ORR), PFS, OS, and biochemical or diagnostic (BOD) PFS. Responses were assessed according to IMWG guidelines; radiographic response assessments were not required [21, 22]. In instances of suspected daratumumab interference with M-protein assessment, a daratumumab-specific reflex assay was used to confirm CRs or sCRs [23]. BOD PFS was defined as the time from the date of randomization to the date of death or to the date of biochemical progression or diagnostic progression, whichever occurred first. Biochemical progression, an exploratory endpoint in SMM, was defined as a measurable increase of ≥25% from nadir value in any of the following during two consecutive visits: serum M-component (absolute increase must be ≥0.5 g/dl), urine M-component (absolute increase must be ≥200 mg/24 h), and, in patients without measurable serum and urine M-protein, the difference between involved and uninvolved FLC levels (absolute increase must be >10 mg/dl; Supplementary Table S1). Diagnostic progression was defined according to SLiM-CRAB criteria plus additional IMWG FLC progression criteria [6, 21]. Specifically, FLC PD was defined as an FLC ratio of ≥100, an involved FLC level of ≥100 mg/l, a ≥25% increase from nadir in the difference between involved and uninvolved FLC levels, and an absolute increase in the difference between involved and uninvolved FLC levels >10mg/dl, as confirmed by two consecutive assessments [6, 21].

Safety evaluations included adverse event (AE) monitoring, physical examinations, electrocardiography, clinical laboratory testing, vital sign measurements, and ECOG performance status assessments using National Cancer Institute Common Terminology Criteria for AEs, Version 4.03 [24].

### Statistical analyses

For each treatment arm, two hypotheses were tested independently: (1) null hypothesis (H_0_): CR rate ≤15%; alternate hypothesis (H_a_): CR rate ≥35% and (2) H_0_: PD/death rate ≥0.346/patient-year (corresponding to a median PFS <24 months); H_a_: PD/death rate ≤0.185/patient-year (median PFS >45 months). We estimated that enrolling 40 patients/arm would provide 90% power to show that the true CR rate is >15% at a one-sided level of 0.05 and 80% power to show that the true PD/death rate is <0.346/patient-year at a one-sided level of 0.1. In addition, if at least two treatment schedules were deemed effective, we estimated that the study would have 85% probability of identifying the arm with the higher CR and 80% probability of identifying the arm with the lower PD/death rate.

PFS and OS were computed by the Kaplan–Meier method.

Additional methods are provided in the Supplementary Information.

## Results

### Patients and treatment

The CENTAURUS study was conducted in 47 sites in Europe, North America, the Middle East, and the Asia Pacific region. A total of 255 patients were screened, of which 132 (51.8%) patients failed screening (Fig. 1b); the most common reason for screening failure was fulfillment of the criteria for active MM [6] (57 patients [43.2% of screen failures]). Between May 1, 2015 and January 2, 2017, all 123 screened patients eligible for the study were randomized, with 41 patients in each arm (intense, intermediate, and short). Baseline demographics and disease characteristics were balanced across the three arms of the study (Table 1). The median age was 61.0 (range 31–81) years, and the median time from SMM diagnosis to enrollment was relatively short, ranging from 5.5 to 7.4 months. One hundred one patients (82.1%) had an ECOG performance status score of 0, while the remaining 22 patients (17.9%) had a score of 1. One hundred patients (81.3%) had at least two risk factors at screening. Ninety patients (73.2%) had disease with an IgG subtype. The median (range) bone marrow PC percentage was 20.0% (10.0–55.0%). The most common cytogenetic abnormalities, del(13q) and gain or amp 1q21, were observed in 16.2% and 20.0% of patients, respectively.

**Table 1:** Baseline demographics and disease characteristics.

	Intense (*n* = 41)	Intermediate (*n* = 41)	Short (*n* = 41)
Median (range) age, years	65.0 (34–79)	62.0 (31–81)	59.0 (39–78)
Female, *n* (%)	24 (58.5)	24 (58.5)	20 (48.8)
Race, *n* (%)			
White	35 (85.4)	37 (90.2)	35 (85.4)
Black or African American	2 (4.9)	1 (2.4)	2 (4.9)
Asian	2 (4.9)	1 (2.4)	1 (2.4)
Other	2 (4.9)	2 (4.9)	3 (7.3)
ECOG performance status score, *n* (%)			
0	32 (78.0)	34 (82.9)	35 (85.4)
1	9 (22.0)	7 (17.1)	6 (14.6)
Risk factors at screening,^a^ *n* (%)			
<2	8 (19.5)	8 (19.5)	7 (17.1)
≥2	33 (80.5)	33 (80.5)	34 (82.9)
Type of disease, *n* (%)			
IgG	33 (80.5)	30 (73.2)	27 (65.9)
IgA	6 (14.6)	7 (17.1)	9 (22.0)
Others	2 (4.9)	4 (9.8)	5 (12.2)
% plasma cells in bone marrow, *n* (%)			
≥10% to <20%	18 (43.9)	17 (41.5)	21 (51.2)
≥20% to <40%	15 (36.6)	17 (41.5)	13 (31.7)
≥40% to <60%	8 (19.5)	7 (17.1)	7 (17.1)
Cytogenetic abnormalities,^b^ *n* (%)			
*n*^c^	37	35	33
t(4;14)	2 (5.4)	3 (8.6)	0
t(14;16)	0	0	0
del(17p)	2 (5.4)	3 (8.6)	1 (3.0)
del(13q)	6 (16.2)	7 (20.0)	4 (12.1)
Gain or amp 1q21	7 (18.9)	6 (17.1)	8 (24.2)
Median (range) time from SMM diagnosis to randomization, months	6.47 (0.4–46.2)	5.52 (0.7–46.7)	7.43 (1.0–56.0)

*ECOG* Eastern Cooperative Oncology Group, *SMM* smoldering multiple myeloma.

^a^Risk factors include abnormal free light chain ratio (<0.126 or >8), serum M-protein ≥3 g/dl, urine M-protein >500 mg/24 h, IgA subtype, and immunoparesis (at least one uninvolved immunoglobulin [IgG, IgA, IgM] decreased >25% below the lower limit of normal) [20].

^b^Cytogenetic abnormalities were detected by FISH and/or karyotyping.

^c^Includes all patients with available cytogenetics data. Among the 105 patients with available cytogenetics data, cytogenetic risk was assessed by karyotyping alone in 13 patients, by FISH alone in 50 patients, and by both karyotyping and FISH in 42 patients.

The prespecified primary analyses for the co-primary endpoints were conducted 6 months (for CR rate) and 12 months (for PD/death rate) after randomization of the last patient; the clinical cutoff dates were February 8, 2017 and August 8, 2017, respectively. At the clinical cutoff date of August 8, 2017, the median duration of follow-up was 15.8 (range 0–23.9) months. In addition, all efficacy and safety endpoints were analyzed after a longer duration of follow-up occurring at the clinical cutoff date of June 29, 2018; for this analysis, the median duration of follow-up was 25.9 (range 0–33.2) months. At the clinical cutoff date of June 29, 2018, the median (range) duration of treatment was 25.8 (1.0–33.1) months in the intense arm, 25.8 (1.9–33.1) months in the intermediate arm, and 1.6 (0.1–1.9) months in the short arm. In the short arm, 38 patients (95.0%) had completed treatment; treatment in the intense and intermediate arms was ongoing at the time of clinical cutoff. The primary reasons for treatment discontinuation were PD (seven patients [5.7%]) and AEs (six patients [4.9%]; Fig. 1). AEs leading to treatment discontinuation included pneumonia, thrombocytopenia, breast disorder, balance disorder, unstable angina, and hypomania (*n* = 1 each). Eight patients (6.5%) discontinued study participation due to patient withdrawal (one, two, and three patients in the intense, intermediate, and short arms, respectively) and death (one patient each in the intermediate and short arms).

### Efficacy

At the primary analysis, CR or better was achieved by one patient (2.4%; 90% confidence interval [CI], 0.1–11.1%) in the intense arm and two patients (4.9%; 90% CI, 0.9–14.6%) in the intermediate arm, with *P* values of 0.9895 and 0.9569, respectively, for testing for the null hypothesis that the CR (sCR + CR) rate was ≤15%. With longer follow-up (clinical cutoff date of June 29, 2018), CR or better was achieved by two patients (4.9%) in the intense arm and four patients (9.8%) in the intermediate arm (Table 2). No patient in the short arm achieved a CR or better at the time of either clinical cutoff. The co-primary endpoint of CR rate >15% was not met at either time point. At the June 29, 2018 clinical cutoff, the ORR was 56.1% (90% CI, 42.1–69.4%) in the intense arm, 53.7% (90% CI, 39.8–67.1%) in the intermediate arm, and 37.5% (90% CI, 24.7–51.7%) in the short arm. Very good partial responses (VGPRs) were achieved by 24.4%, 14.6%, and 17.5% of patients in the intense, intermediate, and short arms, respectively, and partial responses (PRs) were achieved by 26.8%, 29.3%, and 20.0%, respectively.

**Table 2:** Summary of ORR and PD/death rate.^a^

	Intense (*n* = 41)	Intermediate (*n* = 41)	Short (*n* = 41)
ORR summary, *n*^b^	41	41	40^c^
ORR, *n* (%)	23 (56.1)	22 (53.7)	15 (37.5)
90% CI	42.1–69.4	39.8–67.1	24.7–51.7
CR (sCR + CR) rate	2 (4.9)	4 (9.8)	0
*P* value^d^	0.9569	0.7567	
90% CI^e^	(0.9–14.6)	(3.4–21.0)	
sCR	2 (4.9)	3 (7.3)	0
CR	0	1 (2.4)	0
VGPR	10 (24.4)	6 (14.6)	7 (17.5)
PR	11 (26.8)	12 (29.3)	8 (20.0)
SD	18 (43.9)	19 (46.3)	25 (62.5)
PD/death rate summary, *n*^f^	41	41	41
Patients who progressed or died, *n* (%)	5 (12.2)	8 (19.5)	10 (24.4)
Progressed^g^	5 (12.2)	7 (17.1)	10 (24.4)
Died	0	1 (2.4)	1 (2.4)
Total duration of PFS, patient-years	85.2	75.1	66.6
PD/death rate^h^	0.059	0.107	0.150
*P* value^i,j^	<0.0001	<0.0001	<0.0001
80% CI^j^	(0.0251–0.0923)	(0.0583–0.1548)	(0.0893–0.2110)
Biochemical PFS, *n*	41	41	41
Patients who progressed or died, *n* (%)	7 (17.1)	13 (31.7)	25 (61.0)
Median PFS, months (90% CI)	NR (NE–NE)	NR (NE–NE)	15.1 (11.6–23.3)
12-month PFS rate, % (90% CI)	94.9 (84.5–98.4)	77.7 (64.6–86.5)	58.0 (43.6–69.9)
24-month PFS rate, % (90% CI)	84.3 (71.6–91.7)	70.2 (56.5–80.3)	31.5 (19.2–44.6)

*ORR* overall response rate, *PD* progressive disease, *CI* confidence interval, *CR* complete response, *sCR* stringent complete response, *VGPR* very good partial response, *PR* partial response, *SD* stable disease, *PFS* progression-free survival, *NR* not reached, *NE* not estimable, *MM* multiple myeloma, *IMWG* International Myeloma Working Group, *FLC* free light chain, *MRI* magnetic resonance imaging.

^a^Based on the clinical cutoff date of June 29, 2018.

^b^Response rates were assessed in the response-evaluable population (patients who had measurable disease at baseline, received ≥1 dose of study drug, and had ≥1 postbaseline disease assessment).

^c^One patient in the short arm was randomized but did not receive study treatment.

^d^Exact *P* value for testing the null hypothesis that the CR (sCR + CR) rate was ≤15%.

^e^Exact 90% CI.

^f^PD/death rate was assessed in the intent-to-treat population.

^g^PD was defined per the 2014 IMWG diagnostic criteria for MM [6] plus additional IMWG FLC progression criteria (a ≥25% increase from nadir in the difference between involved and uninvolved FLC levels [absolute increase must be >10 mg/dl]) [21]. Most progression events were SLiM-based and consisted primarily of serum FLC ratio ≥100 or more than one focal lesion by MRI. Two progression events, lytic lesions, were CRAB-based.

^h^PD/death rate is the ratio of the patients who progressed or died divided by the total duration of progression-free survival for all patients in patient-years.

^i^*P* value for testing the null hypothesis that the PD/death rate is ≥0.346/patient-year.

^j^Normal approximation.

At the clinical cutoff date of June 29, 2018, five patients (12.2%) in the intense arm, seven patients (17.1%) in the intermediate arm, and ten patients (24.4%) in the short arm had experienced disease progression (Table 2). SLiM-based progression events included involved/uninvolved serum FLC ratio ≥100 (*n* = 13), more than one focal lesion by MRI (*n* = 5), and clonal bone marrow PC percentage ≥60% (*n* = 2); two CRAB-based progression events were reported (lytic lesions), and no fractures related to MM lytic lesions were reported. In the short arm, one patient died due to disease progression, despite the timely initiation of frontline MM therapy (bortezomib/lenalidomide plus radiation).

At the primary analysis, the PD/death rates per patient-year were 0.055 (80% CI, 0.014–0.096) for the intense arm, 0.102 (80% CI, 0.044–0.160) for the intermediate arm, and 0.206 (80% CI, 0.118–0.295) for the short arm. The *P* values for testing the null hypothesis that the PD/death rate per patient-year is ≥0.346 (equivalent to a median PFS <24 months under the exponential distribution) were <0.0001, <0.0001, and 0.0213 for the intense, intermediate, and short arms, respectively. With longer follow-up (clinical cutoff date of June 29, 2018), the PD/death rates per patient-year were 0.059 (80% CI, 0.025–0.092) for the intense arm, 0.107 (80% CI, 0.058–0.155) for the intermediate arm, and 0.150 (80% CI, 0.089–0.211) for the short arm; *P* values for all arms were <0.0001 (Table 2). These data demonstrate that the co-primary endpoint of median PFS ≥24 months was met.

**Fig. 2:**
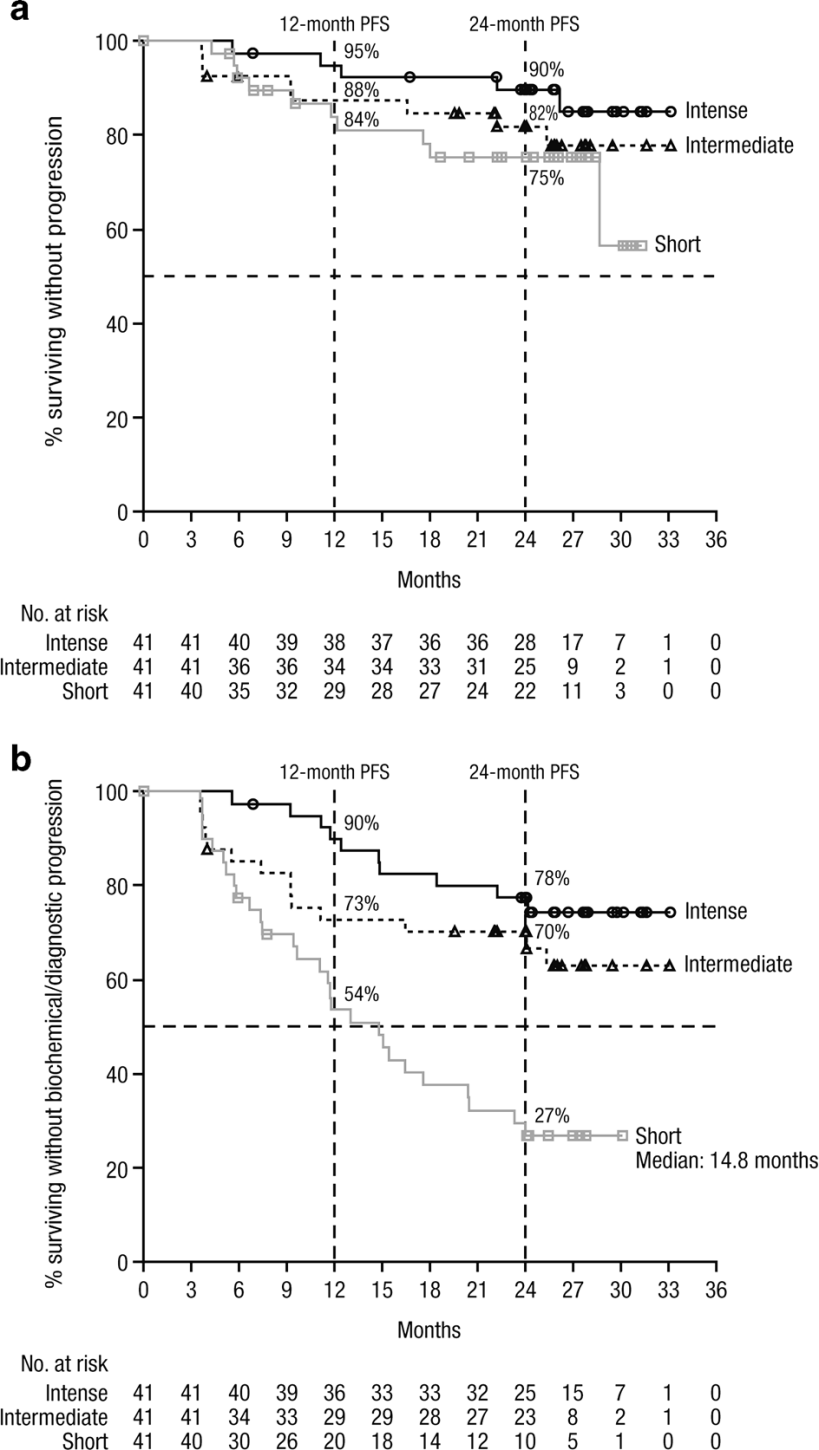
PFS. PFS^a^ with progression defined based **a** on diagnostic^b^ criteria and **b** on biochemical^c^ or diagnostic criteria.^d^ *PFS* progression-free survival, *IMWG* International Myeloma Working Group, *MM* multiple myeloma, *FLC* free light chain. ^a^PFS was assessed in the intent-to-treat population. ^b^Diagnostic progression was defined per the 2014 IMWG criteria for MM [6] plus additional IMWG FLC progression criteria (a ≥25% increase from nadir in the difference between involved and uninvolved FLC levels [absolute increase must be >10 mg/dl]) [21]. ^c^Biochemical progression was defined as a measurable increase of ≥25% from nadir value in any of the following at any point during follow-up: serum M-component (absolute increase must be ≥0.5 g/dl), urine M-component (absolute increase must be ≥200 mg/24 h), and, in patients without measurable serum and urine M-protein, the difference between involved and uninvolved FLC levels (absolute increase must be >10 mg/dl). ^d^Based on the clinical cutoff date of June 29, 2018.

At the clinical cutoff date of June 29, 2018, the median PFS (based on SLiM-CRAB plus IMWG FLC progression criteria) was not reached in any treatment arm (Fig. 2a). The 12-month PFS rates were 95.1% (90% CI, 85.0–98.4%), 87.5% (90% CI, 75.7–93.8%), and 84.0% (90% CI, 71.0–91.5%) for the intense, intermediate, and short arms, respectively, and the 24-month PFS rates were 89.9% (90% CI, 78.5–95.4%), 82.0% (90% CI, 69.0–89.9%), and 75.3% (90% CI, 61.1–85.0%), respectively. The median biochemical PFS was not reached in the intense or intermediate arms and was 15.1 months (90% CI, 11.6–23.3) in the short arm (Table 2). The 12-month biochemical PFS rates were 94.9% (90% CI, 84.5–98.4%), 77.7% (90% CI, 64.6–86.5%), and 58.0% (90% CI, 43.6–69.9%) for the intense, intermediate, and short arms, respectively, and the 24-month rates were 84.3% (90% CI, 71.6–91.7%), 70.2% (90% CI, 56.5–80.3%), and 31.5% (90% CI, 19.2–44.6%), respectively. The median BOD PFS was not reached in the intense or intermediate arms and was 14.8 months (90% CI, 11.1–20.4) in the short arm (Fig. 2b). The 12-month BOD PFS rates were 90.1% (90% CI, 78.8–95.5%), 72.8% (90% CI, 59.2–82.4%), and 53.7% (90% CI, 39.6–65.8%) for the intense, intermediate, and short arms, respectively, and the 24-month BOD PFS rates were 77.5 (90% CI, 64.4–86.4), 70.2 (90% CI, 56.6–80.3), and 26.8 (90% CI, 15.8–39.1), respectively. OS data remain immature; follow-up is ongoing.

### Safety

The safety population comprised 122 patients. At the clinical cutoff date of June 29, 2018, AEs were observed in 100% of patients in the intense and intermediate arms, and 92.5% of patients in the short arm. The most common treatment-emergent AEs (TEAEs) included fatigue, upper respiratory tract infection, cough, insomnia, headache, diarrhea, arthralgia, and nausea (Table 3). No hematologic TEAE occurred in ≥10% of patients in any treatment arm. Grade 3/4 TEAEs were observed in 43.9% of patients in the intense arm, 26.8% of patients in the intermediate arm, and 15.0% of patients in the short arm. The most common grade 3/4 TEAEs (observed in >1 patient in any arm) were hypertension and hyperglycemia (Table 4). No grade 3/4 TEAEs were observed in more than three patients in any arm, and the rate of grade 3/4 infections was ≤5% in all arms. Serious AEs were observed in 31.7% of patients in the intense arm, 14.6% of patients in the intermediate arm, and 10.0% of patients in the short arm (Table 3). Within the first 8 weeks, while all patients were receiving an identical weekly schedule of daratumumab dosing, serious AEs were observed in 12.2%, 0%, and 10.0% of patients in the intense, intermediate, and short arms, respectively. The only serious AE reported in more than one patient in any arm was pneumonia, which was observed in two patients (4.9%) in the intense arm (Table 4). Infusion-related reactions were observed in 56.1%, 43.9%, and 55.0% of patients in the intense, intermediate, and short arms, respectively (Table 3). Most infusion-related reactions were associated with the first infusion. Second primary malignancies were observed in two patients in the intense arm (stage II breast cancer and stage 0 melanoma) and one patient in the intermediate arm (stage I melanoma).

**Table 3:** Safety summary.^a,b^

	Intense (*n* = 41)	Intermediate (*n* = 41)	Short (*n* = 40)^c^
Median (range) duration of treatment, months	25.8 (1.0–33.1)	25.8 (1.9–33.1)	1.6 (0.1–1.9)
Most common (>25%) any grade TEAE, *n* (%)			
Fatigue	17 (41.5)	25 (61.0)	9 (22.5)
Upper respiratory tract infection	15 (36.6)	14 (34.1)	4 (10.0)
Cough	15 (36.6)	13 (31.7)	11 (27.5)
Insomnia	13 (31.7)	13 (31.7)	5 (12.5)
Headache	11 (26.8)	10 (24.4)	13 (32.5)
Diarrhea	11 (26.8)	10 (24.4)	4 (10.0)
Arthralgia	11 (26.8)	9 (22.0)	0
Nausea	8 (19.5)	11 (26.8)	3 (7.5)
Grade 3/4 TEAEs, *n* (%)	18 (43.9)	11 (26.8)	6 (15.0)
Serious AEs, *n* (%)	13 (31.7)	6 (14.6)	4 (10.0)
Within the first 8 weeks	5 (12.2)	0	4 (10.0)
Related to daratumumab	0	1 (2.4)	1 (2.5)
Discontinued treatment due to TEAE, *n* (%)	3 (7.3)	1 (2.4)	2 (5.0)
Related to daratumumab	1 (2.4)^d^	0	1 (2.5)^e^
Any grade IRR rate, *n* (%)	23 (56.1)	18 (43.9)	22 (55.0)

*TEAE* treatment-emergent adverse event, *AE* adverse event, *IRR* infusion-related reaction.

^a^Based on the clinical cutoff date of June 29, 2018.

^b^The safety analysis population included patients who were randomized, received at least one dose of study drug, and contributed any safety data after the start of study treatment.

^c^One patient in the short arm was randomized but did not receive study treatment.

^d^Thrombocytopenia.

^e^Unstable angina.

**Table 4:** Summary of grade 3/4 treatment-emergent adverse events and serious adverse events.^a,b^

	Intense (*n* = 41)		Intermediate (*n* = 41)		Short (*n* = 40)^c^	
Event, *n* (%)	Grade 3/4	Serious	Grade 3/4	Serious	Grade 3/4	Serious
Hypertension	3 (7.3)	0	2 (4.9)	0	1 (2.5)	0
Hyperglycemia	1 (2.4)	1 (2.4)	2 (4.9)	0	0	0
Pneumonia	1 (2.4)	2 (4.9)	1 (2.4)	1 (2.4)	1 (2.5)	1 (2.5)
Increased alanine aminotransferase	1 (2.4)	0	1 (2.4)	0	0	0
Diarrhea	1 (2.4)	0	1 (2.4)	0	0	0
Arthralgia	1 (2.4)	1 (2.4)	0	0	0	0
Atrial fibrillation	1 (2.4)	1 (2.4)	0	0	0	0
Cardiac failure	1 (2.4)	1 (2.4)	0	0	0	0
Dehydration	1 (2.4)	1 (2.4)	0	0	0	0
Hemangioblastoma	1 (2.4)	1 (2.4)	0	0	0	0
Hemiplegia	1 (2.4)	1 (2.4)	0	0	0	0
Hypertriglyceridemia	1 (2.4)	1 (2.4)	0	0	0	0
Leukocytosis	1 (2.4)	1 (2.4)	0	0	0	0
Pericardial effusion	1 (2.4)	1 (2.4)	0	0	0	0
Pulmonary embolism	1 (2.4)	1 (2.4)	0	0	0	0
Streptococcal sepsis	1 (2.4)	1 (2.4)	0	0	0	0
Increased blood pressure	1 (2.4)	0	0	0	0	0
Depression	1 (2.4)	0	0	0	0	0
Dysarthria	1 (2.4)	0	0	0	0	0
Dyspnea	1 (2.4)	0	0	0	0	0
Influenza-like illness	1 (2.4)	0	0	0	0	0
Musculoskeletal disorder	1 (2.4)	0	0	0	0	0
Thrombocytopenia	1 (2.4)	0	0	0	0	0
Hip fracture	0	0	1 (2.4)	1 (2.4)	0	0
Hyponatremia	0	0	1 (2.4)	1 (2.4)	0	0
Intestinal perforation	0	0	1 (2.4)	1 (2.4)	0	0
Osteoarthritis	0	0	1 (2.4)	1 (2.4)	0	0
Pain	0	0	1 (2.4)	1 (2.4)	0	0
Anastomotic leak	0	0	1 (2.4)	0	0	0
Asthma	0	0	1 (2.4)	0	0	0
Cataract	0	0	1 (2.4)	0	0	0
Diverticulitis	0	0	1 (2.4)	0	0	0
Gastrointestinal anastomotic leak	0	0	1 (2.4)	0	0	0
Hypersensitivity vasculitis	0	0	1 (2.4)	0	0	0
Lymphopenia	0	0	1 (2.4)	0	0	0
Angina pectoris	0	0	0	0	1 (2.5)	1 (2.5)
Unstable angina	0	0	0	0	1 (2.5)	1 (2.5)
Sepsis	0	0	0	0	1 (2.5)	1 (2.5)
Abdominal discomfort	0	0	0	0	1 (2.5)	0
Cough	0	0	0	0	1 (2.5)	0
Breast disorder	0	1 (2.4)	0	0	0	0
Embolism	0	1 (2.4)	0	0	0	0
Fall	0	1 (2.4)	0	0	0	0
Pleurisy	0	1 (2.4)	0	0	0	0
Ileus	0	0	0	1 (2.4)	0	0
Malignant melanoma	0	0	0	1 (2.4)	0	0

^a^Based on the clinical cutoff date of June 29, 2018.

^b^The safety analysis population included patients who were randomized, received at least one dose of study drug, and contributed any safety data after the start of study treatment.

^c^One patient in the short arm was randomized but did not receive study treatment.

## Discussion

Currently, SMM is one of few cancer diagnoses where early diagnosis does not mandate early treatment, as there are currently no approved drugs for treatment of this disease. Therefore, patients undergo active monitoring until progression to MM. However, despite rapid advances that continue to be made in MM treatment, including the emergence of chimeric antigen receptor (CAR)-T–based therapies [25], an established curative therapy is not yet available. SMM patients at higher risk for progression to MM may benefit from earlier therapeutic intervention. A need therefore exists for treatments that are both effective and well tolerated in this asymptomatic disease. Regulatory approval of individual agents may present a pathway toward the eventual approval of combination regimens, which may be later accessed by patients who have successfully delayed their progression to MM.

The CENTAURUS study was designed to determine whether daratumumab monotherapy could delay progression from intermediate-risk or high-risk SMM to MM compared with historical observations [19] and to find the optimal schedule of treatment administration. These patients are likely to progress to MM, providing a rationale for evaluating single-agent daratumumab as a therapeutic intervention. Including both intermediate-risk and high-risk patients in this study allowed patients to be recruited within a reasonable timeframe for this proof-of-concept study. In the GEN501 study, a treatment schedule similar to the intense schedule in CENTAURUS demonstrated clinical activity and an acceptable safety profile in patients with RRMM [17]. However, clinical activity was observed even with the abbreviated schedule used in the dose-escalation phase of GEN501 [17], suggesting that the short schedule in CENTAURUS may be sufficient to delay the onset of MM.

The co-primary endpoint of CR rate of >15% was not met at the time of clinical cutoff, which suggests that single-agent daratumumab may not be sufficient to eradicate SMM. However, the PD/death rate and ORR demonstrated that daratumumab does have single-agent activity in SMM. Furthermore, the PFS and BOD PFS data suggest that prolonged dosing of daratumumab monotherapy delays both diagnostic progression and biochemical progression compared with short-term dosing.

Three studies have elaborated on the importance of biomarkers evolving during follow-up [26–28]. Evolving changes in M-protein and hemoglobin levels and evolving differences in FLC were found to be risk factors for progression. Studies are ongoing to determine how treatment should be guided based on the predictive value of these biomarkers.

A retrospective analysis of 206 patients with SMM demonstrated that patients who experience biochemical progression are likely to experience clinical progression to MM [27]. The median time from recognition of evolving type (progressive increase in serum M-protein until development of MM) to progression to MM was 1.1 years, and the 3-year progression rate was 71%, indicating that detection of the evolving patterns within the first year after diagnosis of SMM allows identification of patients at high risk for progression who are therefore candidates for early therapeutic intervention. In the current study, differences in BOD PFS among the three treatment arms were more pronounced than those in PFS, which may suggest that incorporation of biochemical progression into the criteria for progression of SMM to MM may be important. While the current study was not designed to demonstrate that biochemical progression should be incorporated into these criteria, our results provide a platform for future hypothesis-generating analyses.

The safety profile of daratumumab monotherapy in patients with SMM was acceptable and was consistent with those observed in RRMM studies [17, 18]. It should be noted that the intense and intermediate arms had longer treatment durations and therefore extended AE collecting periods compared with the short arm, both of which may have contributed to the differences observed in AE incidences between treatment arms. Although the rates of grade 3/4 and serious AEs were highest in the intense arm, these rates (43.9% and 31.7%, respectively) were similar to those observed in a comparable population of patients with high-risk SMM treated with placebo (grade ≥3 AE: 33%; serious AE: 31%) [19]. In addition, the number of patients discontinuing treatment due to AEs was similarly low across treatment arms.

Currently, two main approaches to SMM treatment are being investigated: eradication vs. control [29]. Several eradication-based studies have been conducted in patients with SMM. In a pilot study of carfilzomib plus Rd with lenalidomide extension in high-risk SMM, among the 11 of 12 patients who completed eight treatment cycles, all achieved VGPR or better [30]. Minimal residual disease–negative status was detected in 11 patients by multiparametric flow cytometry and in nine patients by next-generation sequencing. The regimen was deemed tolerable, with one patient discontinuing treatment due to grade 3 congestive heart failure. In a phase 2 study, patients with high-risk SMM received carfilzomib plus Rd as induction before, and as consolidation after, high-dose melphalan plus autologous stem-cell transplantation, followed by Rd maintenance [31]. Among patients who had undergone transplantation and were evaluable at 3 months (*n* = 29), the ORR was 100% (sCR, CR, and VGPR rates were 65%, 3%, and 21%, respectively), and the minimal residual disease–negative rate was 58%. No patient discontinued treatment due to treatment-related AEs. A control-based strategy was examined in a phase 3 study of Rd followed by lenalidomide maintenance vs. observation in 119 patients with high-risk SMM. The median time to progression was not reached vs. 21 months, and the 3-year survival rate was 94 vs. 80% [7]. The ORR was 79% during the induction phase (*n* = 57), including four sCRs, four CRs, and six VGPRs, and increased to 90% during the maintenance phase (*n* = 50). A randomized phase 3 study in patients with high-risk SMM showed that lenalidomide monotherapy vs. observation improved PFS (hazard ratio, 0.28; 95% CI, 0.12–0.63; *P* *=* 0.0005) after a median follow-up of 35 months; the 12- and 24-month PFS rates were 98% and 93% for the lenalidomide group, respectively, and 89% and 76% for the observation group. The ORR was 48.9% in the lenalidomide group and 0% in the observation group, and the rate of VGPR or better was 4.4% in the lenalidomide group compared with 0% in the observation group [32].

Previous daratumumab population pharmacokinetic analyses in MM indicated that a concentration of 236 μg/ml would be sufficient to maintain 99% model-predicted target saturation [33]. Pharmacokinetic data from the current study indicate that most patients in the intense arm maintain trough (predose) concentrations near or above this target saturation concentration throughout the weekly, every-2-week, and every-4-week dosing periods (Supplementary Information). However, in the intermediate arm, mean trough concentrations fell below the target saturation concentration by the end of the first cycle of every-8-week dosing (Cycle 3 Day 1) and, in the short arm, concentrations fell below this concentration by 8 weeks after the last dose. These data are consistent with those obtained previously with daratumumab in patients with MM, and the maintenance of effective trough concentrations likely contributed to less biochemical progression [33–35].

Treating a disease that has not yet manifested clinically is a concept that may face challenges, especially with health funding systems. However, the potential risks of early intervention should be balanced with the comorbidities, diminished quality of life, and higher risk for death associated with MM. Rapid advancements continue to be made in MM therapy, and some of these new therapies may translate into development of earlier treatments for SMM.

In CENTAURUS, approximately half of the screened patients were excluded; most (43.2%) of the screening failures were attributed to having active MM. As the definition of high-risk SMM has continued to evolve [1], and based on the need to improve stratification based on disease risk, the inclusion criteria were further revised for the phase 3 AQUILA (NCT03301220) study, which is testing subcutaneous administration of daratumumab (which may improve patient convenience) vs. active monitoring in SMM. In addition, the efficacy and pharmacokinetic data support extended daratumumab dosing over the shortened 8-week dosing schedule and informed the dosing schedule used in the AQUILA study. Finally, while quality of life assessments were not performed in CENTAURUS, patient-reported outcomes will be evaluated in AQUILA.

In conclusion, the findings of this study suggest that daratumumab has single-agent activity and demonstrates acceptable tolerability in intermediate-risk and high-risk SMM.

## Supplementary information

Supplementary Information for Landgren et al

## References

[1] Rajkumar SV, Landgren O, Mateos MV (2015). Smoldering multiple myeloma. Blood.

[2] Landgren O, Kyle RA, Pfeiffer RM, Katzmann JA, Caporaso NE, Hayes RB (2009). Monoclonal gammopathy of undetermined significance (MGUS) consistently precedes multiple myeloma: a prospective study. Blood.

[3] Dispenzieri A, Kyle R, Katzmann JA, Therneau TM, Larson D, Benson J (2008). Immunoglobulin free light chain ratio is an independent risk factor for progression of smoldering (asymptomatic) multiple myeloma. Blood.

[4] Lakshman A, Rajkumar SV, Buadi FK, Binder M, Gertz MA, Lacy MQ (2018). Risk stratification of smoldering multiple myeloma incorporating revised IMWG diagnostic criteria. Blood Cancer J.

[5] Perez-Persona E, Vidriales MB, Mateo G, Garcia-Sanz R, Mateos M-V, Garcia de Coca A (2007). New criteria to identify risk of progression in monoclonal gammopathy of uncertain significance and smoldering multiple myeloma based on multiparameter flow cytometry analysis of bone marrow plasma cells. Blood.

[6] Rajkumar SV, Dimopoulos MA, Palumbo A, Blade J, Merlini G, Mateos MV (2014). International Myeloma Working Group updated criteria for the diagnosis of multiple myeloma. Lancet Oncol.

[7] Mateos MV, Hernandez MT, Giraldo P, de la Rubia J, de Arriba F, Lopez Corral L (2013). Lenalidomide plus dexamethasone for high-risk smoldering multiple myeloma. N Engl J Med.

[8] Mateos MV, Hernandez MT, Giraldo P, de la Rubia J, de Arriba F, Corral LL (2016). Lenalidomide plus dexamethasone versus observation in patients with high-risk smouldering multiple myeloma (QuiRedex): long-term follow-up of a randomised, controlled, phase 3 trial. Lancet Oncol.

[9] Lin P, Owens R, Tricot G, Wilson CS (2004). Flow cytometric immunophenotypic analysis of 306 cases of multiple myeloma. Am J Clin Pathol.

[10] Santonocito AM, Consoli U, Bagnato S, Milone G, Palumbo GA, Di Raimondo F (2004). Flow cytometric detection of aneuploid CD38(++) plasmacells and CD19(+) B-lymphocytes in bone marrow, peripheral blood and PBSC harvest in multiple myeloma patients. Leuk Res.

[11] de Weers M, Tai YT, van der Veer MS, Bakker JM, Vink T, Jacobs DC (2011). Daratumumab, a novel therapeutic human CD38 monoclonal antibody, induces killing of multiple myeloma and other hematological tumors. J Immunol.

[12] Lammerts van Bueren J, Jakobs D, Kaldenhoven N, Roza M, Hiddingh S, Meesters J (2014). Direct in vitro comparison of daratumumab with surrogate analogs of CD38 antibodies MOR03087, SAR650984 and Ab79. Blood.

[13] Overdijk MB, Verploegen S, Bogels M, van Egmond M, Lammerts van Bueren JJ, Mutis T (2015). Antibody-mediated phagocytosis contributes to the anti-tumor activity of the therapeutic antibody daratumumab in lymphoma and multiple myeloma. MAbs.

[14] van de Donk NWCJ, Janmaat ML, Mutis T, Lammerts van Bueren JJ, Ahmadi T, Sasser AK (2016). Monoclonal antibodies targeting CD38 in hematological malignancies and beyond. Immunol Rev.

[15] Krejcik J, Casneuf T, Nijhof IS, Verbist B, Bald J, Plesner T (2016). Daratumumab depletes CD38^+^ immune-regulatory cells, promotes T-cell expansion, and skews T-cell repertoire in multiple myeloma. Blood.

[16] Overdijk MB, Jansen JH, Nederend M, Lammerts van Bueren JJ, Groen RW, Parren PW (2016). The therapeutic CD38 monoclonal antibody daratumumab induces programmed cell death via Fcgamma receptor-mediated cross-linking. J Immunol.

[17] Lokhorst HM, Plesner T, Laubach JP, Nahi H, Gimsing P, Hansson M (2015). Targeting CD38 with daratumumab monotherapy in multiple myeloma. N Engl J Med.

[18] Lonial S, Weiss BM, Usmani S, Singhal S, Chari A, Bahlis N (2016). Daratumumab monotherapy in patients with treatment-refractory multiple myeloma (SIRIUS): an open-label, randomised, phase 2 trial. Lancet.

[19] Brighton TA, Khot A, Harrison SJ, Ghez D, Weiss BM, Kirsch A (2019). Randomized, double-blind, placebo-controlled, multicenter study of siltuximab in high-risk smoldering multiple myeloma. Clin Cancer Res.

[20] Dispenzieri A, Stewart AK, Chanan-Khan A, Rajkumar SV, Kyle RA, Fonseca R (2013). Smoldering multiple myeloma requiring treatment: time for a new definition?. Blood.

[21] Durie BGM, Harousseau JL, Miguel JS, Blade J, Barlogie B, Anderson K (2006). International uniform response criteria for multiple myeloma. Leukemia.

[22] Rajkumar SV, Harousseau JL, Durie B, Anderson KC, Dimopoulos M, Kyle R (2011). Consensus recommendations for the uniform reporting of clinical trials: report of the International Myeloma Workshop Consensus Panel 1. Blood.

[23] McCudden C, Axel AE, Slaets D, Dejoie T, Clemens PL, Frans S (2016). Monitoring multiple myeloma patients treated with daratumumab: teasing out monoclonal antibody interference. Clin Chem Lab Med.

[24] US Department of Health and Human Services, National Institutes of Health, National Cancer Institute. Common Terminology Criteria for Adverse Events (CTCAE). Version 4.03. https://evs.nci.nih.gov/ftp1/CTCAE/CTCAE_4.03/CTCAE_4.03_2010-06-14_QuickReference_5x7.pdf. Accessed 4 Jan 2019.

[25] Ghosh A, Mailankody S, Giralt SA, Landgren CO, Smith EL, Brentjens RJ (2018). CAR T cell therapy for multiple myeloma: where are we now and where are we headed?. Leuk Lymphoma.

[26] Ravi P, Kumar S, Larsen JT, Gonsalves W, Buadi F, Lacy MQ (2016). Evolving changes in disease biomarkers and risk of early progression in smoldering multiple myeloma. Blood Cancer J.

[27] Fernandez de Larrea C, Isola I, Pereira A, Cibeira MT, Magnano L, Tovar N (2018). Evolving M-protein pattern in patients with smoldering multiple myeloma: impact on early progression. Leukemia.

[28] Wu V, Moshier E, Leng S, Barlogie B, Cho HJ, Jagannath S (2018). Risk stratification of smoldering multiple myeloma: predictive value of free light chains and group-based trajectory modeling. Blood Adv.

[29] Landgren O (2017). Shall we treat smoldering multiple myeloma in the near future?. Hematol Am Soc Hematol Educ Program.

[30] Korde N, Roschewski M, Zingone A, Kwok M, Manasanch EE, Bhutani M (2015). Treatment with carfilzomib-lenalidomide-dexamethasone with lenalidomide extension in patients with smoldering or newly diagnosed multiple myeloma. JAMA Oncol.

[31] Mateos MV, Martinez Lopez J, Rodriguez-Otero P, Ocio EM, Gonzalez MS, Oriol A (2017). Curative strategy for high-risk smoldering myeloma (GEM-CESAR): carfilzomib, lenalidomide and dexamethasone (KRd) as induction followed by HDT-ASCT, consolidation with Krd and maintenance with Rd. Blood.

[32] Lonial S, Jacobus SJ, Weiss M, Kumar S, Orlowski RZ, Kaufman JL (2019). E3A06: Randomized phase III trial of lenalidomide versus observation alone in patients with asymptomatic high-risk smoldering multiple myeloma. J Clin Oncol.

[33] Xu XS, Yan X, Puchalski T, Lonial S, Lokhorst HM, Voorhees PM (2017). Clinical implications of complex pharmacokinetics for daratumumab dose regimen in patients with relapsed/refractory multiple myeloma. Clin Pharm Ther.

[34] Clemens PL, Yan X, Lokhorst HM, Lonial S, Losic N, Khan I (2016). Pharmacokinetics of daratumumab following intravenous infusion in relapsed or refractory multiple myeloma after prior proteasome inhibitor and immunomodulatory drug treatment. Clin Pharmacokinet.

[35] Yan X, Clemens PL, Puchalski T, Lonial S, Lokhorst HM, Orlowski RZ (2015). Target-mediated drug disposition of daratumumab following intravenous infusion in relapsed or refractory multiple myeloma after prior proteasome inhibitors and immunomodulatory drugs: a population pharmacokinetic analysis. Blood.

