# Supplementary material for: Daratumumab monotherapy for patients with intermediate-risk or high-risk smoldering multiple myeloma: a randomized, open-label, multicenter, phase 2 study (CENTAURUS)
Source: Leukemia. 2020 Feb 5;34(7):1840–52. doi: 10.1038/s41375-020-0718-z (PMC7326703; doi:10.1038/s41375-020-0718-z)
Supplement: Supplementary file 1 — Supplementary Information for Landgren et al [file 41375_2020_718_MOESM1_ESM.docx]

**Supplementary Information for Landgren et al. Daratumumab Monotherapy for Patients With Intermediate-risk or High-risk Smoldering Multiple Myeloma: A Randomized, Open-label, Multicenter, Phase 2 Study (CENTAURUS).**

**SUPPLEMENTARY METHODS**

*Risk factors for progression to multiple myeloma (MM)*

Risk factors included abnormal free light chain (FLC) ratio (<0.126 or >8), serum M-protein ≥3 g/dl, urine M-protein >500 mg/24 h, IgA subtype, and immunoparesis (at least 1 uninvolved immunoglobulin [IgG, IgA, IgM] decreased >25% below the lower limit of normal).[1]

*Management of infusion-related reactions and infections*

To mitigate infusion-related reactions, patients received methylprednisolone 60 to 100 mg, acetaminophen 650 to 1 000 mg, and diphenhydramine 25 to 50 mg before each daratumumab infusion; montelukast 10 mg approximately 1 hour prior to daratumumab infusion was optional. To prevent delayed infusion-related reactions, methylprednisolone 20 mg was administered on the 2 days after daratumumab infusions in Cycle 1. Supportive management for infections, including vaccination with pneumococcal vaccine before initiating daratumumab treatment, was strongly recommended.

*FLC progression criteria*

The International Myeloma Working Group (IMWG) FLC diagnostic criteria are designed for de novo diagnosis rather than for detecting progression of smoldering multiple myeloma to multiple myeloma. Daratumumab may preferentially reduce the uninvolved light chain, therefore suggesting progression to multiple myeloma based on these criteria despite actual therapeutic response/absolute reduction of the involved light chain. To avoid overcalling progression based on FLC ratio, FLC progression was assessed based on modified FLC criteria that combine IMWG FLC progression criteria[2] with IMWG FLC diagnostic criteria[3], all of which had to be met during 2 consecutive visits: involved/uninvolved serum FLC ratio ≥100, involved FLC ≥100 mg/l, and ≥25% increase from lowest value (nadir) in the difference between involved and uninvolved FLC levels (with an absolute increase of >10 mg/dl).

*Radiographic assessments*

Skeletal radiography or low-dose computed tomography (CT) was performed every 12 months and at biochemical progression or suspected disease progression. For patients with negative magnetic resonance imaging (MRI) results at baseline, an M-protein increase of ≤25%, and no other signs of clinical progression, only MRI was performed, and low-dose whole-body CT or skeletal survey was performed only when focal lesions were identified. An MRI of the spine and pelvis was performed every 6 months for the first 3 years, followed by every 12 months, and at biochemical progression or suspected disease progression. Whole-body MRI was conducted per local practice or when clinically indicated.

*Pharmacokinetic and immunogenicity assessments*

Venous blood samples (5 ml/sample) were collected from all patients to assess both the serum concentration (pharmacokinetics) of daratumumab and the presence of anti-daratumumab antibodies (immunogenicity) using validated immunoassay methods. The pharmacokinetic parameters assessed included C_min_, defined as the minimum observed daratumumab concentration prior to the start of the infusion, and C_max_, defined as the maximum observed daratumumab concentration at the end of the daratumumab infusion. All patients who received at least 1 daratumumab infusion and had at least 1 pharmacokinetic sample concentration value after the first infusion were included in the pharmacokinetics analysis set. For the immunogenicity assessments, serum samples were screened for antibodies binding to daratumumab, and serum titer was also determined from confirmed positive samples. The incidence of anti-daratumumab antibodies was summarized for all patients who received at least 1 daratumumab infusion and had at least 1 sample collected after the start of the first daratumumab infusion for detection of anti-daratumumab antibodies.

**Supplementary Table S1. Diagnostic and Biochemical Progression Criteria**

| **Type of Progression** | **Criteria** |
| --- | --- |
| Diagnostic Progression (IMWG diagnostic criteria for MM[3] plus additional IMWG FLC progression criteria[2]) | Clonal bone marrow plasma cells ≥10% or biopsy-proven bony or extramedullary plasmacytoma,^a^ plus ≥1 of the following:   - Calcium elevation (>0.25 mmol/l [>1 mg/dl] higher than the ULN or >2.75 mmol/l [>11 mg/dl]) - Renal insufficiency (creatinine clearance^b^ <40 ml/min or serum creatinine >177 µmol/l [>2 mg/dl]) - Anemia (hemoglobin <10 g/dl [<6.5 mmol/l] or >2 g/dl [>1.25 mmol/l] lower than the LLN) - Bone disease (≥1 osteolytic lesion on skeletal radiography, CT, or PET-CT^c^) - Clonal bone marrow plasma cell percentage^a^ ≥60% - Involved/uninvolved serum FLC ratio^d^ ≥100 - ≥25% increase from nadir in the difference between involved and uninvolved FLC levels (absolute increase must be >10 mg/dl) - >1 focal lesion^e^ on MRI studies |
| Biochemical progression | A measurable increase of ≥25% from nadir value in any of the following at any point during follow-up:   - Serum M-component (absolute increase must be ≥0.5 g/dl) - Urine M-component (absolute increase must be ≥200 mg/24 h) - In patients without measurable serum and urine M-protein, the difference between involved and uninvolved FLC levels (absolute increase must be >10 mg/dl) |

IMWG, International Myeloma Working Group; MM, multiple myeloma; FLC, free light chain; ULN, upper limit of normal; LLN, lower limit of normal; CT, computed tomography; PET, positron emission tomography; MRI, magnetic resonance imaging.

^a^Clonality should be established by showing κ/λ light-chain restriction on flow cytometry, immunohistochemistry, or immunofluorescence. Bone marrow plasma cell percentage should preferably be estimated from a core biopsy specimen; in case of a disparity between the aspirate and the core biopsy, the highest value should be used.

^b^Measured or estimated by validated equations.

^c^If bone marrow has <10% clonal plasma cells, >1 bone lesion is required to distinguish from solitary plasmacytoma with minimal marrow involvement.

^d^These values are based on the serum Freelite assay (The Binding Site Group, Birmingham, UK). The involved FLC must be ≥100 mg/l.

^e^Each focal lesion must be ≥5 mm in size.

**PHARMACOKINETICS AND IMMUNOGENICITY DATA**

**Supplementary Figure S1** shows the mean (standard deviation [SD]) peak (end of infusion) and trough (predose) concentrations of daratumumab during treatment and 4 and 8 weeks post treatment. After the first daratumumab infusion (Cycle 1 Day 1), the mean (SD) C_max_ was similar across the 3 treatment arms (386.93 [71.0] μg/ml, 385.96 [82.6] μg/ml, and 389.16 [67.4] μg/ml in the intense, intermediate, and short arms, respectively). The peak and trough concentrations of daratumumab on Cycle 1 Day 50 revealed that concentrations remained similar through the first cycle, when patients in all arms received the same dose of daratumumab. Daratumumab accumulation during the first 9 doses resulted in a 2.8- to 2.9-fold increase in C_max­_ in the intense and intermediate arms, with mean (SD) serum concentrations of 1 089.29 (220.0) μg/ml and 1 134.46 (308.4) μg/ml, respectively, at Cycle 2 Day 1. The mean (SD) Cycle 2 Day 1 trough concentration after 8 weekly doses was 663.12 (201.0) μg/ml and 702.31 (245.5) μg/ml in the intense arm and intermediate arm, respectively. By the end of Cycle 2, the mean (SD) trough concentration was higher in the intense arm (456.76 [219.1] μg/ml) than in the intermediate arm (230.04 [185.7] μg/ml). The serum daratumumab concentration in the short arm was 172.87 (157.6) μg/ml 8 weeks after the last daratumumab dose. At the end of every-4-week dosing in the intense arm (Cycle 8 Day 1), the mean (SD) C_min­_ decreased to 168.78 (110.2) μg/ml; at the same timepoint for the intermediate arm (after 6 cycles of every-8-week dosing), the mean (SD) C_min_ was notably lower (27.95 [24.9] μg/ml). Moderate interpatient variability for daratumumab exposure was observed. Of the 120 patients who had suitable samples for immunogenicity testing, none were positive for anti-daratumumab antibodies.

**Supplementary Figure S1. Mean serum peak and trough concentrations of daratumumab.**


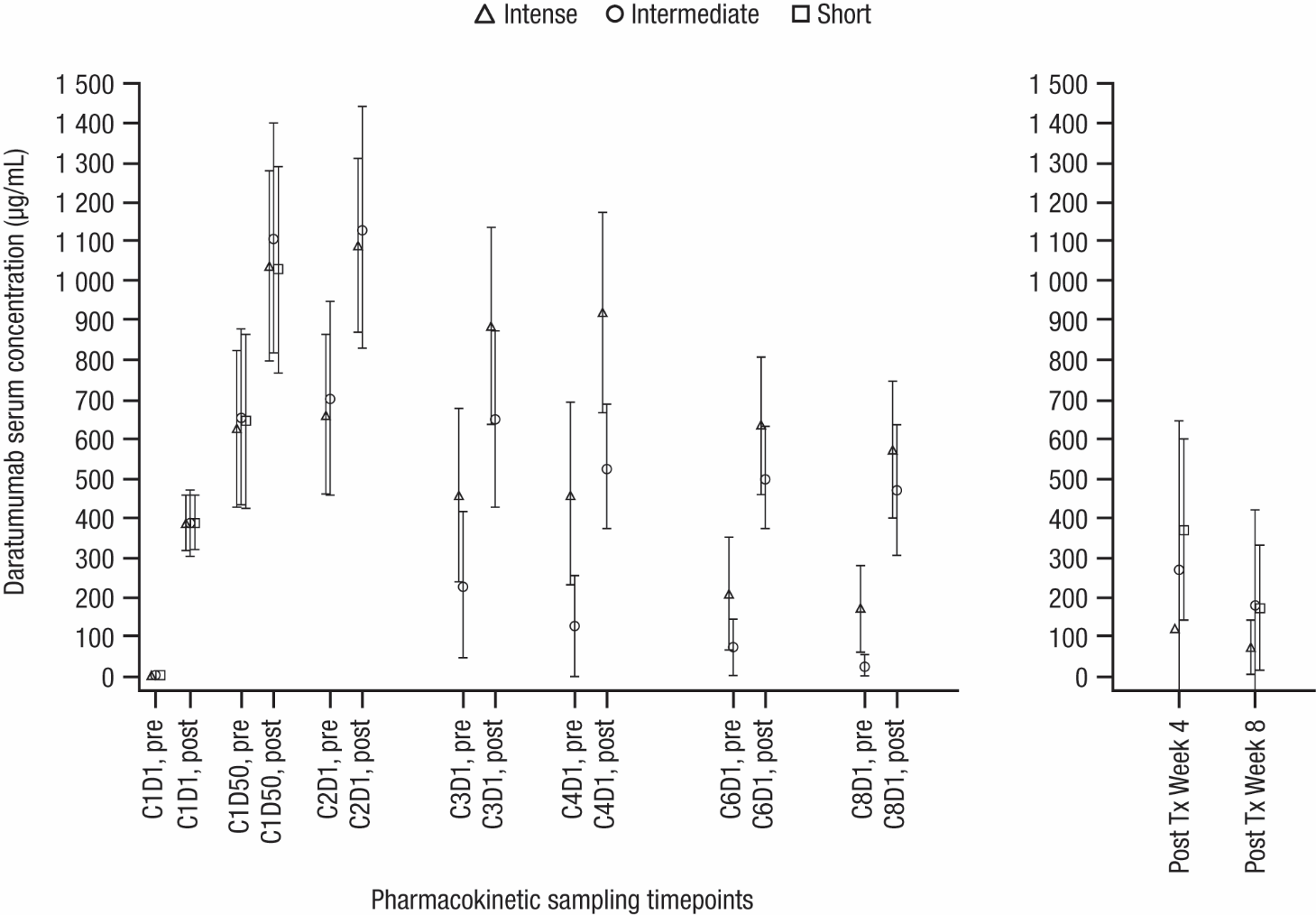


Predose samples with a time of collection after the start of infusion and postdose samples with a time of collection >20 minutes before the end of infusion or before the start of infusion were excluded from the summary statistics. Error bars represent mean ± standard deviation.

C, Cycle; D, Day; pre, preinfusion; post, postinfusion; Tx, treatment.

**REFERENCES**

1. Dispenzieri A, Stewart AK, Chanan-Khan A, Rajkumar SV, Kyle RA, Fonseca R, et al. Smoldering multiple myeloma requiring treatment: time for a new definition? Blood 2013; 122: 4172–4181.

2. Durie BGM, Harousseau JL, Miguel JS, Blade J, Barlogie B, Anderson K, et al. International uniform response criteria for multiple myeloma. Leukemia 2006; 20: 1467–1473.

3. Rajkumar SV, Dimopoulos MA, Palumbo A, Blade J, Merlini G, Mateos MV, et al. International Myeloma Working Group updated criteria for the diagnosis of multiple myeloma. Lancet Oncol 2014; 15: e538–e548.
